# Two-stage closed sinus lift: a new surgical technique for maxillary sinus floor augmentation

**DOI:** 10.1007/s10561-015-9505-x

**Published:** 2015-03-10

**Authors:** Kornel Krasny, Marta Krasny, Artur Kamiński

**Affiliations:** 1 Medicare Dental Practice, ul. Popiełuszki 17a/102, 01-595 Warsaw, Poland; 2 Department of Orthodontics, Medical University of Warsaw, ul. Nowogrodzka 59, 01-005 Warsaw, Poland; 3 Department of Transplantology and Central Tissue Bank, Warsaw Medical University, Warsaw, Poland

**Keywords:** Maxillary sinus floor augmentation, Allograft, Alveolar ridge augmentation, Dental implants

## Abstract

Bone tissue atrophy may constitute a relative contraindication for implantation. The methods used in reconstruction of the alveolar ridge within the lateral section of the maxilla have been well known but not perfect. Presentation of the two-stage, closed sinus lift technique as well as efficacy evaluation of reconstruction of the alveolar ridge in the maxilla within its vertical dimension with the use of this technique. The total procedure was performed in 26 out of 28 patients qualified for the study. The height of the alveolar ridge at the site of the planned implantation was no <3 mm, the width of the ridge was no <5 mm. During the treatment stage 1 the sinus lift was performed for the first time. The created hollow was filled with allogeneic granulate. After 3–6 months stage 2 was performed consisting in another sinus lift with simultaneous implantation. The treatment was completed with prosthetic restoration after 6 months of osteointegration. In 24 out of 26 cases stage 1 was completed with the average ridge height of 7.2 mm. In stage 2, simultaneously with the second sinus lift, 26 implants were placed and no cases of sinusitis were found. In the follow-up period none of the implants were lost. The presented method is efficient and combines the benefits of the open technique—allowing treatment in cases of larger reduction of the vertical dimension and the closed technique—as it does not require opening of the maxillary sinus.

## Introduction

The height of the alveolar ridge in the maxilla is the resultant of masticatory forces transferred by the periodontal ligament system to the bone and pneumatisation of maxillary sinuses beginning with eruption of the third molars (Misch 1999). Bone atrophy in the maxilla is a physiological process, which accelerates in case of tooth extractions (Sorni´ et al. 2005). In females higher post-extraction bone resorption is observed compared to males (Sağlam 2002), which may be related to density of the bone tissue and hormonal balance of the body. The research proves that more severe atrophy may be expected when molars are extracted rather than premolars (Wehrbein and Diedrich 1992) and when a greater number of adjacent teeth are extracted (Sharan and Madjar 2008). Prolonged healing time resulting from numerous and traumatic extractions also promotes more severe atrophy of bone tissue (Sharan and Madjar 2008). Unskilful tooth extraction may be associated with the damage of the thin lamina dividing the maxillary sinus and the alveolus as well as rupture of the sinus-lining membrane, which hence exacerbates the extraction-related, physiological atrophy of the ridge hard tissue.

Insufficient vertical dimension of the alveolar ridge is a relative contraindication for implantation. Owing to techniques of alveolar ridge reconstruction introduced in surgery in 1970s the optimal size of the ridge bone may be restored (Sorni´ et al. 2005; Schwartz-Arad et al. 2004) and implantation may be successfully performed (Levin et al. 2004). The first to be described was the open technique, which allowed performing the procedure in patients with a ridge of at least 4 mm (Balaji 2013); however, successful attempts were made in more severe reduction of the vertical dimension (Chaushu et al. 2009). If the atrophy of the vertical dimension of the alveolar ridge is less severe, closed techniques are used. Their advantages include lower invasivity and single-stage sinus lift combined with implant embedment. However, in view of limited visibility within the operative field and greater initial dimension of the alveolar ridge (7 mm), the planned range of augmentation must be smaller (Pal et al. 2012).

Limitations of the techniques mentioned above inspire clinicians to seek new methods of ridge reconstruction in the lateral segment of the maxilla before implantation, which would allow combination of the advantages of open sinus lift with the low risk of the closed sinus lift.

## Objective of the study

Presentation of two-stage closed sinus lift and evaluation of this new technique in maxillary alveolar ridge reconstruction within its vertical dimension.

## Materials and methods

**Fig. 1:**
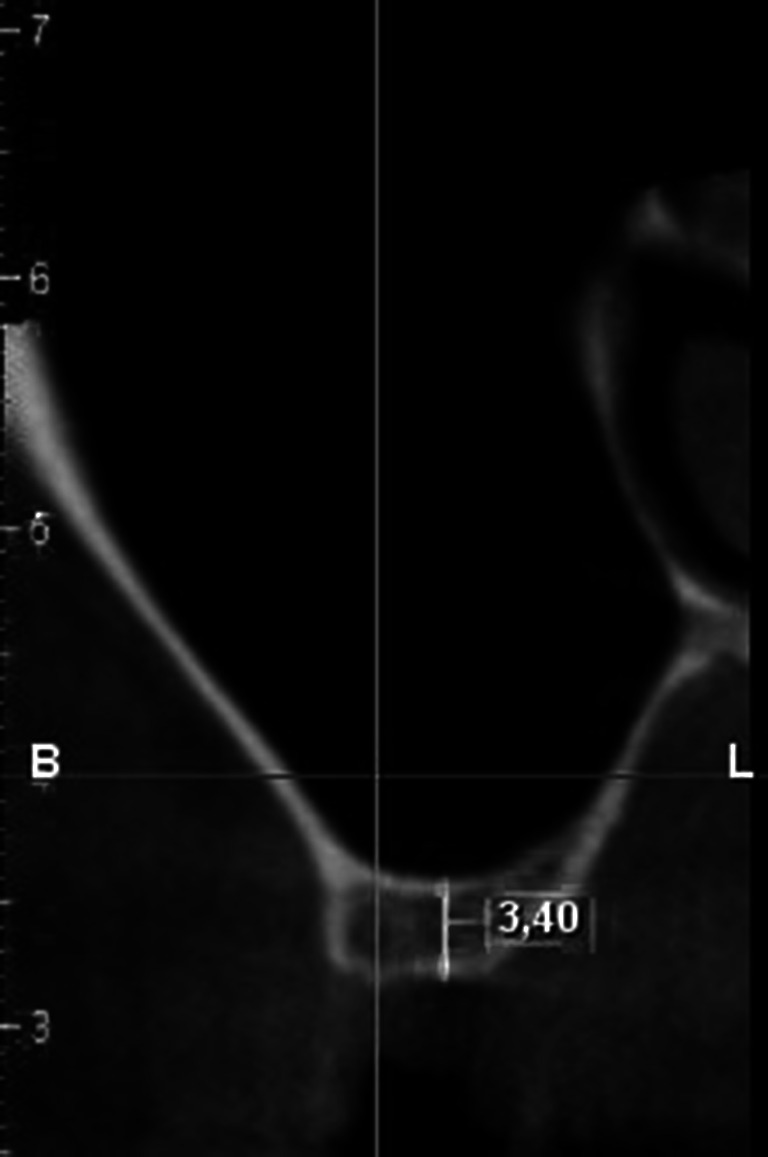
CT scan before grafting

The technique of two-stage sinus lift was used in 28 subjects aged 29–66 (mean age: 44) who had reported to have a dental defect restored with implant insertion. Before treatment computed tomography of the maxilla (Fig. 1) was performed in all the patients. Inclusion criteria comprised no inflammation within the sinus on the side of the dental defect, minimum height of the alveolar ridge within the implantation area: 3 mm, minimum width of the ridge: 5 mm (thus no necessity for widening procedure), lack of general diseases.

### Stage 1

Under local anaesthesia with 4 % Ubistesin forte an incision was made at the top of the alveolar ridge from the palatal side within the toothless gap. The cut was extended perpendicularly to the ridge, across periodontium of the teeth adjacent to the gap and further on to the oral vestibule.

After the mucoperiosteal flap was detached normal bone tissue was found. With a spot drill the optimal place was marked for future intraosseous implantation. Then, with a guide drill a hollow was made 1 mm shallower than the height of the alveolar ridge within this area previously calculated based on CT. Subsequently the sinus floor was augmented for the first time with a chisel kit for sinus lifts and a surgical hammer. Owing to the concave shape of the upper part of the chisel, bone shavings were obtained and the sinus floor was shifted inwards. Additionally, the other working part of the chisel of slightly conical shape caused concentration of bone tissue within the lateral walls of the tunnel. In order to reduce the force needed to push the bone with next chisels, the outer lamina dura was removed with an implant drill 1 number bigger than the next chisel.

**Fig. 2:**
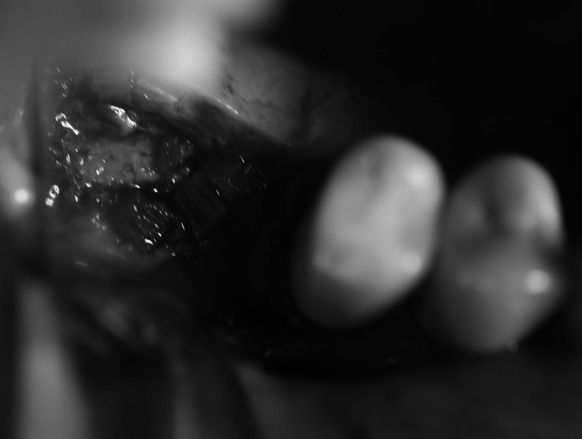
Allograft extending the height of the ridge during stage 1

The last chisel used to elevate the sinus floor was one size wider than the expected diameter of the implant to be embedded. Maxillary sinus floor elevation was done in stages with the use of subsequent chisels. In order to reduce the risk of rupture of the Schneiderian membrane or bone chip dislocation, chiselling was performed very slowly and carefully so dilation of the bone canal progressed gradually. Continuity of the mucous membrane was verified on numerous occasions intraoperatively with a sinus probe ended with a ball. The elevated sinus floor was fixed and filled with the patient’s own bone shifted from the alveolar ridge, whereas the bone void of conical shape with a cut apex was filled with frozen, radiation sterilised allogeneic bone obtained from the Tissue Bank (Fig. 2).

The procedure was finished by extending the mucoperiosteal flap obtained with cut peritoneum which was then repositioned and fixed with mattress sutures. Postoperatively an antibiotic, anaelgesic, and anti-oedemic treatment was administered and mouth rinsing with an antiseptic and surgical site protection was recommended.

The treatment stage 1 was finished when the sutures were removed following 2 and 6 weeks after the procedure healing of the tissues was investigated. Temporary prosthetic restorations were also examined for pressure exerted on the surgical sites.

### Stage 2

**Fig. 3:**
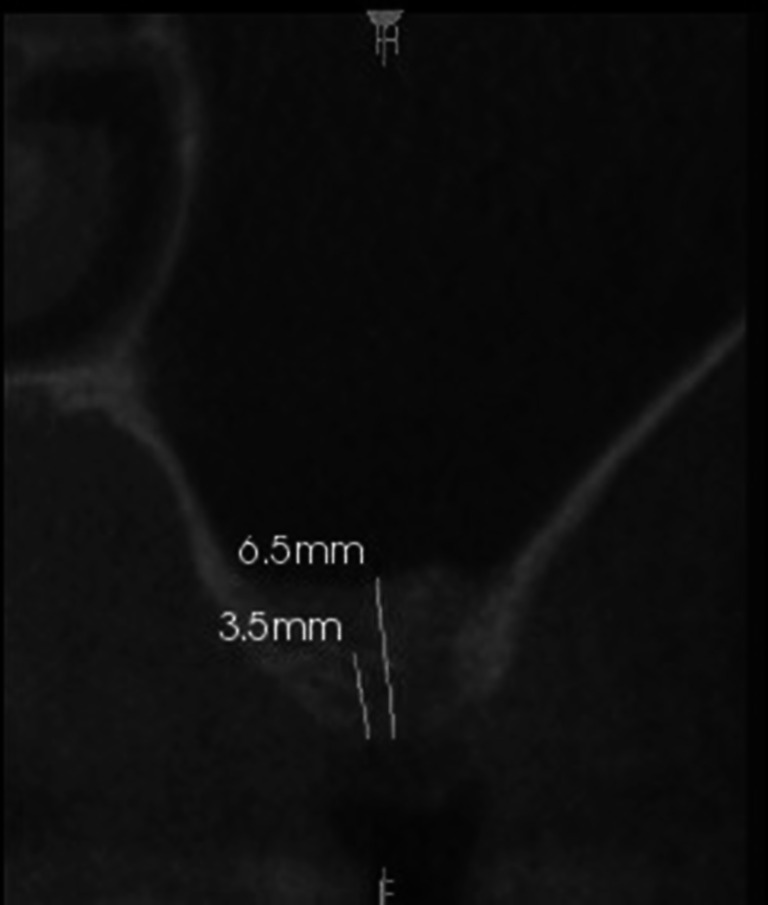
CT scan after stage 1 of the two-stage closed sinus lift

After 3–6 months, before the next stage of surgical treatment, when no inflammation was found within the adjacent tissues and the sinus, a follow-up CT was performed (Fig. 3).

**Fig. 4:**
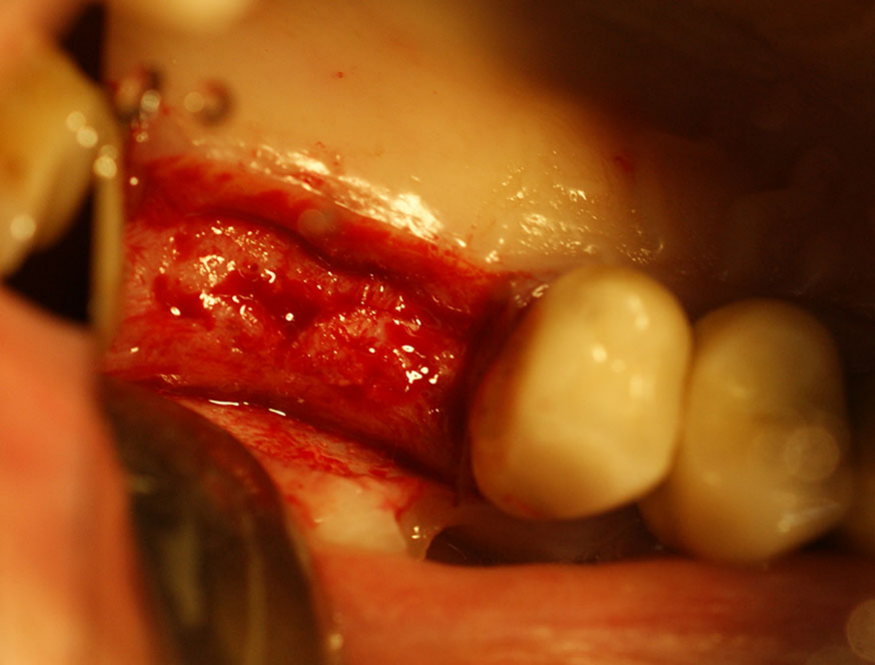
Healed alveolar ridge after treatment stage 1

Under local anaesthesia an incision of the mucous membrane was done just like in stage 1. After the flap was detached and bone structure was evaluated (Fig. 4), the procedure of the alveolar ridge drilling and gradual sinus floor elevation with a chisel kit was repeated. The only difference was that the last chisel to be used was the same diameter as the planned implant. The resulting bone void was filled with the embedded implant which after obtaining primary stability and covering with the flap was sutured without drainage. The patient received the same recommendations as in stage 1.

The sutures were removed after 2 weeks. Treatment stage 2 was finished with a 6-month osteointegration period.

**Fig. 5:**
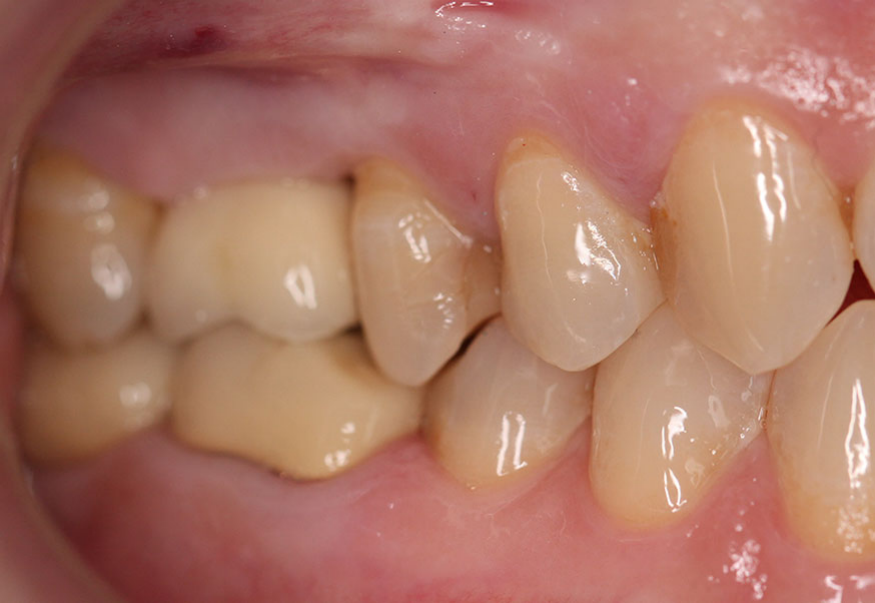
Two and a half years after the two-stage sinus lift was completed

Stage 3 comprised implant-supported prosthetic restoration (Fig. 5).

## Results

The two-stage sinus lift was performed in 28 patients. Two subjects initially qualified for the procedure did not report for continuation of treatment after stage 1. In the other two cases stage 1 was unsuccessful (the required increase of the ridge height was not obtained), most probably due to a rupture of the Schneiderian membrane and partial resorption of the graft. In those cases standard closed sinus lift was performed and a shorter implant was embedded during stage 2. Twenty-six implants BIOMET 3I were embedded in regenerated bone tissue.

In three subjects partial separation of the wound edges was found, which healed by granulation. As the incision line did not cross the surgical site, the graft was not revealed and no major complications were caused. In one case pressure of the prosthetic restoration exerted on the surgical site was observed which was corrected at a follow-up visit after 2 weeks of the procedure. No symptoms of inflammation in the sinuses were found in any of the cases, including those with unsuccessful stage 1.

The mean, maximum, and minimum primary height of the alveolar process, the growth of bone tissue after stage 1 measured in CT as well as the height of the ridge before implantation were presented in Table 1. The augmentation areas and length of the implants are presented in Table 2.

**Table 1:** Graft healing time, initial height of the ridge, sinus lift at stage 1, and ridge height after stage 1

	Min	Max	Mean
Initial height of the ridge	3 mm	5.6 mm	4.22 mm
Sinus lift at stage 1	2.4 mm	4.9 mm	3.94 mm
Ridge height after stage 1	6.5 mm	8.9 mm	7.6 mm
Graft 1 healing time	2.5 months	11 months	5.4 months

**Table 2:** Number of procedures and the length of implants depending on the area of surgery

	Number of procedures within the area	Implant length		
		10 mm	8.5 mm	8 mm
Area of the first molar	14	4	10	
Are of the second premolar	10	1	4	4
Area of the first premolar	4	1	2	

**Fig. 6:**
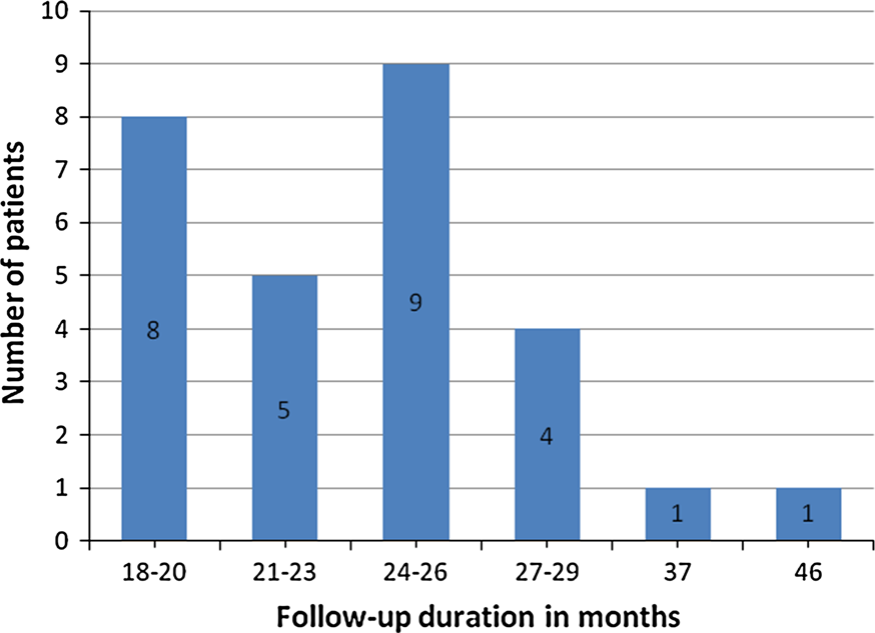
Duration of patients’ follow-up expressed in months

During the follow-up period, the visual of which is presented in Fig. 6, no loss of stability was found in any of the implants. In two cases the prosthetic crown had been partially loosened, which was easily corrected by tightening with a torque wrench.

## Discussion

The choice of a technique for bone augmentation depends mostly on the initial height of the ridge at the site of future implantation. If the thickness of the bone does not provide primary stability of the implant and is <5 mm (Valentini et al. 2000), the method providing good and predictable outcomes is the procedure of open sinus lift. However, it constitutes a burden for the patient as it interferes with the sinus and bears a higher risk of infection (Schwartz-Arad et al. 2004; Balaji 2013), in smokers in particular (Barone et al. 2006). The advantage of this technique is that it allows restoration of a severely reduced ridge (Balaji 2013). However, studies proved that it was better to insert a shorter implant and perform a closed sinus lift than risk an open sinus lift to embed a longer implant (Esposito et al. 2010). Authors of this article had similar experiences; therefore in order to reduce the failure risk the closed sinus lift was performed twice with the doctor’s own modification, which despite unfavourable conditions for simultaneous implant embedment, provided good outcomes in two stages. There have been also reports on successful ridge reconstruction with 1 mm of the patient’s own bone (Winter and Pollack 2002).

The most important disadvantage of the closed sinus lift is the risk of a rupture of the sinus lining membrane; therefore some clinicians use an inflatable device or fill the void with augmentation material before the wall of the sinus is forced into (Stelzle and Benner 2011). Studies by Hernandez-Alfaro demonstrated that implant survival depends on the size of perforation (Hernández-Alfaro et al. 2008). In the discussed study on one hand the two-stage sinus lift prevented excessive straining of the Schneiderian membrane and allowed good primary stability of the implant during its embedment combined with the second sinus lift. On the other hand it allowed regeneration of the bone tissue to a greater extent (the mean of 3.94 mm in stage 1), which in the two-stage procedure provided better outcomes than the traditional method suggested by Summers (Summers 1994). Despite multiple verifications of continuity of the sinus-lining membrane as well as better visibility within the operative field, we failed to avoid the membrane rupture in two cases. There have been also reports of no influence of the membrane rupture on the success of the closed sinus lift (Ardekian et al. 2006; Karabuda et al. 2006).

An autograft is commonly believed to be the best material for reconstruction. However, this choice is associated with the necessity to harvest the graft from the area of the mentum or the retromolar pad or extraoral locations, when a larger amount of the graft is needed. Another surgical site is associated with increased number of possible complications (Guarnieri et al. 2006; Ewers 2005), which makes the patients dissatisfied. There have been reports on lack of advantage of autografts over allogeneic materials (Valentini et al. 2000; Del Fabbro et al. 2004), and even reports on high susceptibility of autografts to resorption (Wallace 2003), reaching as much as 49.5 % after 6 months of the procedure (Ewers 2005). Similar implant survival following the use of bone substitutes and autografts inspires clinicians to choose the former (Valentini et al. 2000), and osteoinductive and osteoconductive properties of allografts (Hallman et al. 2005) lead to the choice of the material in this study.

The latest research reported that the primary stability of the implant depended on the diameter of the implant rather than its length (Maiorana et al. 2014). Good outcomes during a 10-year follow-up were obtained when using 8 mm implants (Mangano et al. 2014). Alternatively, short implants may be used of efficacy comparable to those of standard length (Al-Hashedi et al. 2014), as it happened in two cases when the procedure was modified due to the failure of stage 1. However, the expected time of exploitation of those implants remains unknown (Esposito et al. 2010). The studies reported that in the lateral segment of the maxilla the preferred length of the implant amounted to 6–10 mm (Tutak et al. 2013). In case of severe atrophy an open sinus lift was necessary to embed implants of this size. The suggested technique seemed to be worth considering as there was no interference with the lumen of the sinus.

A disadvantage related to the procedure described above is the longer time between commencement of reconstruction and delivery of the prosthetic restoration compared to a standard sinus lift procedure. The minimum of 3 months is needed after stage 1 for the graft to reorganise and replace it with the patient’s own bone in order to obtain normal primary stability of the implant during stage 2. However, when using the open technique this period is longer, reaching nearly 1.5 years as graft healing and reorganisation in these cases takes 6–9 months, i.e. it is longer than the time suggested in this paper. Not before this period is finished the implants can be embedded and they may not be weighed down with prostheses until another 6 months pass (Davarpanah et al. 2003).

## Conclusions

The discussed technique of the two-stage sinus lift was an efficient method for reconstruction of atrophied alveolar ridge with the initial ridge height of 3 mm with no necessary opening of the maxillary sinus.

The technique had the advantages of the closed sinus lift, i.e. lower risk of infection within the sinus and the advantages of the open technique, i.e. more extended reconstruction of the ridge when its vertical dimension is severely reduced. It allowed ridge expansion up to approximately 5 mm only in stage 1.

A smaller initial height of the ridge (3 mm) compared to the conventional closed method, provided good control over the procedure owing to better visibility within the operative field.

Two-stage, delayed surgical treatment extended the time of the entire dental defect restoration procedure up to 13 months, which must be clearly explained to the patient before the treatment is started.

Employing drills for removal of the lamina dura of the alveolar ridge before the chisels are used for dilating the bone reduced negative sensations of the patient during the sinus lift and did not provoke negative disposition of the patient towards subsequent treatment stages.
